# MethCNA: a database for integrating genomic and epigenomic data in human cancer

**DOI:** 10.1186/s12864-018-4525-0

**Published:** 2018-02-13

**Authors:** Gaofeng Deng, Jian Yang, Qing Zhang, Zhi-Xiong Xiao, Haoyang Cai

**Affiliations:** 1 Center of Growth, Metabolism, and Aging, Key Laboratory of Bio-Resources and Eco-Environment, College of Life Sciences, Sichuan University, Chengdu, Sichuan 610064 China; 2 Jiangsu Center for the Collaboration and Innovation of Cancer Biotherapy, Xuzhou, Jiangsu 221002 China; 3 Cancer Institute, Xuzhou Medical University, Xuzhou, Jiangsu 221002 China

**Keywords:** Copy number aberration, DNA methylation, Data integration, Cancer, Infinium HumanMethylation450 BeadChip, Genomic data, Epigenomic data

## Abstract

**Background:**

The integration of DNA methylation and copy number alteration data promises to provide valuable insight into the underlying molecular mechanisms responsible for cancer initiation and progression. However, the generation and processing of these datasets are costly and time-consuming if carried out separately. The Illumina Infinium HumanMethylation450 BeadChip, initially designed for the evaluation of DNA methylation levels, allows copy number variant calling using bioinformatics tools.

**Results:**

A substantial amount of Infinium HumanMethylation450 data across various cancer types has been accumulated in recent years and is a valuable resource for large-scale data analysis. Here we present MethCNA, a comprehensive database for genomic and epigenomic data integration in human cancer. In the current release, MethCNA contains about 10,000 tumor samples representing 37 cancer types. All raw array data were collected from The Cancer Genome Atlas and NCBI Gene Expression Omnibus database and analyzed using a pipeline that integrated multiple computational resources and tools. The normalized copy number aberration data and DNA methylation alterations were obtained. We provide a user-friendly web-interface for data mining and visualization.

**Conclusions:**

The Illumina Infinium HumanMethylation450 BeadChip enables the interrogation and integration of both genomic and epigenomic data from exactly the same DNA specimen, and thus can aid in distinguishing driver from passenger mutations in cancer. We expect MethCNA will enable researchers to explore DNA methylation and copy number alteration patterns, identify key oncogenic drivers in cancer, and assist in the development of targeted therapies. MethCNA is publicly available online at http://cgma.scu.edu.cn/MethCNA.

**Electronic supplementary material:**

The online version of this article (10.1186/s12864-018-4525-0) contains supplementary material, which is available to authorized users.

## Background

Genomic instability is a hallmark of malignant tumors, causing DNA copy number changes in most cancer types [1, 2]. These copy number aberrations (CNAs) are important influential factors for altered gene expression levels in cancer [3]. Genomic alterations may confer growth advantage of cancer cells and are usually associated with poor prognosis. Recurrent CNA occurs in multiple tumor samples across the same genomic region and plays crucial roles in tumorigenesis [4]. The characterization of CNAs in various cancer types has lead to identification of a large number of genes that contribute to cancer initiation and progression [5–7].

In addition to copy number aberrations, DNA methylation is an important regulator of gene transcription, and is one of the most studied epigenetic modifications [8]. The methylated cytosines are almost exclusively located in CpG dinucleotide sequences [9]. CpGs are uniformly distributed across the genome, and some of them are concentrated in short regions named CpG islands. Methylation in CpG islands within gene promoters usually leads to gene silencing. Association of altered DNA methylation patterns of the promoter CpG islands with the expression profile of cancer genes has been found in many tumor types [10–12]. Aberrant hypomethylation may induce genome instability and overexpression of oncogenes, while hypermethylation in promoter regions of tumor suppressor genes may perturb cell cycle regulation, apoptosis and DNA repair, and result in malignant cellular transformation [13]. Therefore, DNA methylation status may serve as an epigenetic biomarker for cancer diagnosis and prognosis, and has been studied extensively in human cancer.

The integration of different ‘omics’ data types is an increasingly important approach for understanding the fundamental mechanisms of cancer development. Genomic and epigenomic alterations are key regulators of gene expression, and may act in concert to drive tumorigenesis and promote progression towards a malignant phenotype [14]. Genes that are both amplified and hypomethylated or deleted and hypermethylated are likely to play a key role in cancer development. DNA methylation and copy number alteration data from the same tumor specimen may facilitate to elucidate the synergistic mechanisms for the inactivation of tumor suppressor genes or the activation of oncogenic pathways [6, 15]. Copy number aberration profiles and DNA methylation patterns can be measured genome-wide with microarrays. Although at present arrays offer the most cost-effective solutions for producing genomic and epigenomic data, the costs remains a major concern for the large-scale assessment of multiple datasets. Moreover, the computational burden and storage requirements are increased substantially if these data are generated on separate array platforms.

The Illumina Infinium HumanMethylation450 BeadChip (450 k) is based on similar biochemical reaction principle and technology as the single nucleotide polymorphism (SNP) arrays, and is able to robustly detect CNAs as a no-cost byproduct of methylation studies [16, 17]. A variety of bioinformatics tools have been developed for copy number calling from the 450 k methylation array. The detection of copy number and methylation alterations in a single experiment is particularly important when considering the potential effects of tumor heterogeneity on cancer development [17]. The subtle areas of a tumor may be genetically and epigenetically different, and thus confer a different phenotypic trait, such as differing metastatic potential. In recent years, a growing number of studies have generated genome-wide DNA methylation profiles of thousands of cancer samples using Infinium HumanMethylation450 platform, including The Cancer Genome Atlas (TCGA) project, which represents one of the largest efforts to systematically characterize the molecular profiles of cancers [18]. These data are valuable resources for meta-analysis and may provide insight into molecular mechanisms underlying tumorigenesis. However, the processing and integration of these datasets are laborious and time-consuming.

Here we present MethCNA, a comprehensive database for the integrated analysis of DNA methylation and copy number alterations in human cancer. Currently, the database contains about 10,000 publicly available tumor samples interrogated by Infinium HumanMethylation450 BeadChip. The raw array data were collected from TCGA and NCBI Gene Expression Omnibus (GEO) database [19] and processed through a pipeline that integrates several widely used Illumina Infinium 450 k array analysis tools. We developed a user-friendly web-interface and online tools for data access, analysis and visualization. ‘Omics’ data integration and exploration hold great promise for the identification of novel cancer genes, and will enable the development and selection of optimal therapies targeting driver aberrations. MethCNA is designed to meet the growing interest in integrating ‘omics’ data, and is a resource for large-scale integration analysis of genomic and epigenomic data in human cancer.

## Construction and content

### Data source for MethCNA

MethCNA includes genome-wide copy number alterations and DNA methylation profiles, which were simultaneously interrogated by the Illumina Infinium HumanMethylation450 BeadChip. Over 10,000 publicly available tumor samples were collected from TCGA and GEO database. Our data selection criteria are that (1) the tumor samples must be assayed by Illumina Infinium HumanMethylation450 BeadChip; (2) the raw signal intensity data (.IDAT) files must be downloadable for re-analysis and (3) the tumor type matched tissue-specific normal samples are available as a reference for data analysis. In the current release of MethCNA, we collected 28 and 30 data series from TCGA and GEO, respectively. These data series consist of 9964 tumor samples across 37 cancer types. Cancers were defined by their histological types and sites of origin. To provide standardized information on cancer types, International Classification of Diseases for Oncology, 3rd Edition (ICD-O-3) morphology and topography terms and codes were assigned to each tumor sample (Additional file 1: Table S1 and Additional file 2: Table S2). For five tumor types in TCGA (Brain Lower Grade Glioma [LGG], Acute Myeloid Leukemia [LAML], Lymphoid Neoplasm Diffuse Large B-cell Lymphoma [DLBC], Mesothelioma [MESO], Testicular Germ Cell Tumors [TGCT]), the normal samples could not be obtained, and thus are not included in our database. Detailed statistics of cancer types and samples are shown in Table 1.

**Table 1:** Summary of array data contained in MethCNA

Cancer type	TCGA		GEO	
	Sample	Series	Sample	Series
Acute myeloid leukemia	0	0	88	3
Adrenocortical carcinoma	80	1	0	0
Atypical teratoid rhabdoid tumor	0	0	150	1
Bladder urothelial carcinoma	419	1	0	0
Breast invasive carcinoma	796	1	316	3
Cervical cancer	309	1	0	0
Cholangiocarcinoma	36	1	0	0
Colon adenocarcinoma	316	1	104	2
Diffuse intrinsic pontine glioma	0	0	25	1
Esophageal carcinoma	186	1	0	0
Glioblastoma multiforme	153	1	72	1
Head and neck squamous cell carcinoma	530	1	0	0
Kidney Chromophobe	65	1	0	0
Kidney renal clear cell carcinoma	325	1	46	1
Kidney renal papillary cell carcinoma	276	1	0	0
Liver hepatocellular carcinoma	380	1	0	0
Lung adenocarcinoma	475	1	164	1
Lung squamous cell carcinoma	370	1	0	0
Medulloblastoma	0	0	119	2
Neuroblastoma	0	0	32	1
Oligodendroglioma	0	0	46	1
Ovarian adenocarcinoma	10	1	345	3
Pancreatic adenocarcinoma	185	1	167	1
Pheochromocytoma and Paraganglioma	184	1	0	0
Prostate adenocarcinoma	503	1	20	3
Rectum adenocarcinoma	99	1	6	1
Sarcoma	265	1	0	0
Schwannoma	0	0	40	1
Skin cutaneous melanoma	473	1	160	2
Small cell lung carcinoma	0	0	11	1
Squamous cell skin carcinoma	0	0	7	1
Stomach adenocarcinoma	396	1	0	0
Thymoma	124	1	0	0
Thyroid carcinoma	515	1	0	0
Uterine carcinosarcoma	57	1	0	0
Uterine corpus endometrial carcinoma	439	1	0	0
Uveal melanoma	80	1	0	0
Total	8046	28	1918	30

### Data processing pipeline

**Fig. 1:**
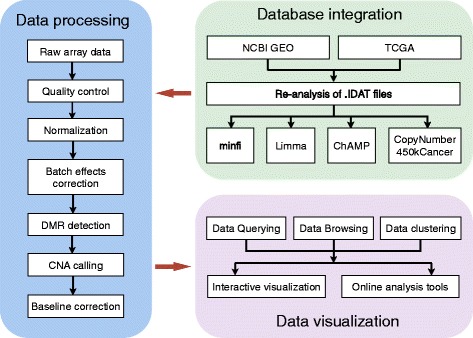
The schematic overview of the data processing pipeline. It consists of three major modules. The raw array data are processed with a series of bioinformatics tools, and the normalized data are used for visualization and analysis

To reanalyze the raw array data, we downloaded signal intensity data (.IDAT) files from public microarray repositories. The data processing pipeline integrates a collection of widely used R packages, including minfi [20], limma [21], ChAMP [22] and CopyNumber450kCancer [23]. A schematic overview of the data processing pipeline is shown in Fig. 1. For all these packages, we used the default settings of the tools. The data files were processed at a per data series level. For each data series, the raw array files were run through the pipeline in batch mode, which integrates several analysis steps from preprocessing (e.g. normalization and batch effect analysis) to basic analysis (e.g. copy number aberrations calling and detection of differentially methylated regions) as well as tool for cancer-specific analysis (e.g. baseline correction for accurate copy number calling). The Bioconductor package minfi was used to perform quality control and generate normalized beta values [20]. The Subset-quantile within array normalization (SWAN) [24] and Beta-Mixture Quantile (BMIQ) [25] normalization methods were employed. The differentially methylated probes and regions were identified by limma package [21]. Benjamini-Hochberg method was used for *p*-value adjustment. For detecting copy number alterations from the Illumina 450 k array platform, we employed the R package ChAMP, which infers copy number changes using intensity of individual and surrounding probes [22]. We used the Beta-value method for differential methylation analysis, since the Beta-value has an intuitive biological interpretation [26]. However, for data series that less than 30 samples, we employed M-value method for the analysis, because in the limit of small sample size, M-values allow more reliable identification of true positives [27]. In order to optimize CNA calling, we integrated the CopyNumber450kCancer R package into the pipeline for baseline estimation and correction using the maximum density peak estimation (MDPE) method [23]. For TCGA datasets, we performed batch effect reduction by ComBat [28] method in the sva R package [29]. We mainly focused on two covariates, namely tissue source sites (TSS), which may introduce bias during sample preparation, and Slides, which may cause bias in data generation process. We treated Slide as batch variable and TSS as covariate. For cases that TSS are confounded with Slide, we only used Slide as batch variable for the correction. This correction was applied to both beta values and signal intensities, which were used for differentially methylated regions calling and copy number alterations calling, respectively. Batch correction was followed by manual inspection of quality control plots generated by ChAMP to evaluate the correction performance.

For TCGA datasets, the tumor type abbreviations were assigned as data series IDs, and the TCGA barcodes were extracted from the annotations file and used as sample IDs. A full list of sample and series IDs, cancer types and related publications is maintained on the MethCNA website. These information can also be found in Additional file 1: Table S1 and Additional file 2: Table S2. For gene level analysis, the standard gene names and locations were downloaded from the UCSC Genome Browser FTP server [30]. All genome coordinates were based on human genome assembly NCBI Build 37/hg19. Since cancer-related DNA methylation studies concentrate on different biologically relevant genome regions, we provide several gene and CpG island regions for data analysis, including promoter, transcriptional start site (TSS), untranslated region (UTR), exon, gene body, CpG island, shore and shelf. The genomic annotations of these functional regions were obtained from the UCSC Genome Browser [30].

### Database architecture and implementation details

The normalized DNA methylation data at both the probe and gene level, and the called copy number states (gain, loss or neutral) of the segmentation data were stored in a MySQL database (version 5.5.49). We developed a user-friendly web interface for users to query and visualize the processed array data. The main functions of MethCNA include search, browse and clustering analysis. The web server runs on a dedicated Linux machine with the Apache HTTP server version 2.4.7. The web application used PHP (version 5.5.9) and HTML to serve web pages. The client-side interactive user interface was designed using JavaScript libraries and jQuery plugins, and the ggplot2 R package [31] and self-written R scripts were used for data visualization. The website has been tested to ensure functionality across different operating systems and browsers, including Internet Explorer, Safari, Firefox, Opera and Chrome.

## Utility and discussion

### Data querying

**Fig. 2:**
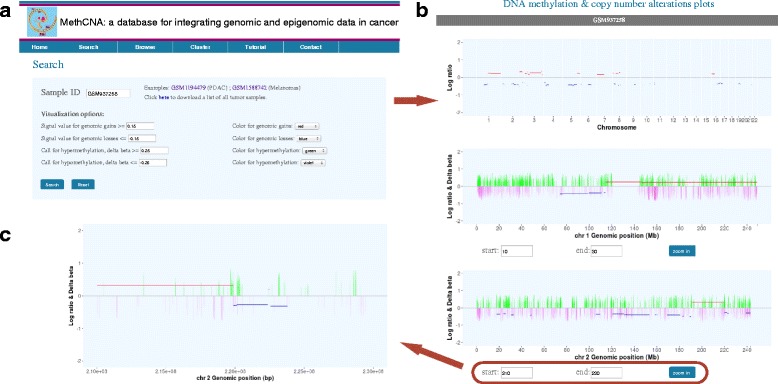
The web interface for query by sample. **a** A set of parameters and thresholds can be used for customized data visualization. **b** The result page displays both copy number aberrations and differentially methylated positions. **c** Areas of interest can be zoomed in for more detail

MethCNA contains 58 data series consisting of 9964 tumor samples across 37 cancer types. The ‘Search’ page provides two options for users to explore data of interest: query by array ID and query by gene. For query by ID, GEO array accession number or TCGA barcode can be input directly into the query box to retrieve copy number and methylation data of the corresponding array. The entire list of array IDs and related information is provided on MethCNA web page. The search interface offers a set of parameters or thresholds for customized data visualization (Fig. 2a). The thresholds for signal value represent cut-offs from which a segment is considered a genomic gain or loss. The delta beta values are used to calculate the percentage methylation difference between cancer and normal tissue. The positive and negative values of delta beta correspond to relative hyper- and hypomethylation, respectively. The empirical optimal thresholds for the Infinium 450 K platform are displayed as defaults. A set of colors can be used to intuitively display both types of data in the same figure. The results page shows CNAs and differentially methylated positions (DMP) for each chromosome (Fig. 2b). The interactive visualization interface allows users to zoom-in to an area of interest by inputting the start and stop genomic locations (Fig. 2c). This will facilitate to identify potential genes that are affected by both CNAs and DNA methylation.

**Fig. 3:**
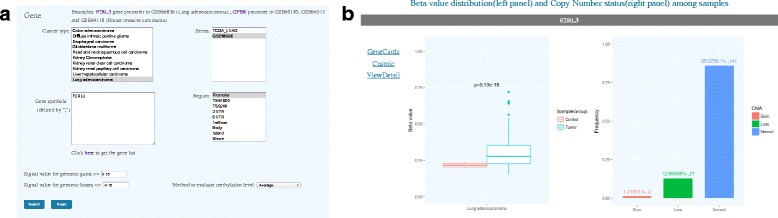
The web interface for query by gene. **a** The interface to select cancer type, data series and gene regions. **b** The differential methylation status and the frequency of genomic imbalances of a gene are illustrated by boxplot and histogram, respectively

The second option is query by genes, which allows users access to gene-specific aberration information in selected datasets (Fig. 3a). Cancer types and data series can be chosen in the list box. The data series field supports multiple selections, and the field content changes dynamically according to the selected cancer type. Gene names can be input in the text field, and multiple gene names may be specified separated with a semicolon. The standard gene names and symbols can be downloaded from MethCNA web page. The thresholds for defining genomic gains and losses are also provided. There are seven gene regions and four CpG island (CGI) regions that can be selected for analysis. The gene promoter region is assigned as default. If genetic and epigenetic variant data is available for the inquired genes, the results page shows the differential methylation status of each gene in selected tumors by boxplot, and the frequency of genomic gains and losses by histogram (Fig. 3b). The ‘View Detail’ link opens a new page to show detailed alteration data about the gene of interest in each sample. For further information of gene annotations, the result page provides links to the corresponding entries of GeneCards [32] and Catalogue of Somatic Mutations in Cancer (COSMIC) databases [33]. This feature will allow researchers to identify promising genetic and epigenetic biomarkers in human cancer.

### Data browsing

**Fig. 4:**
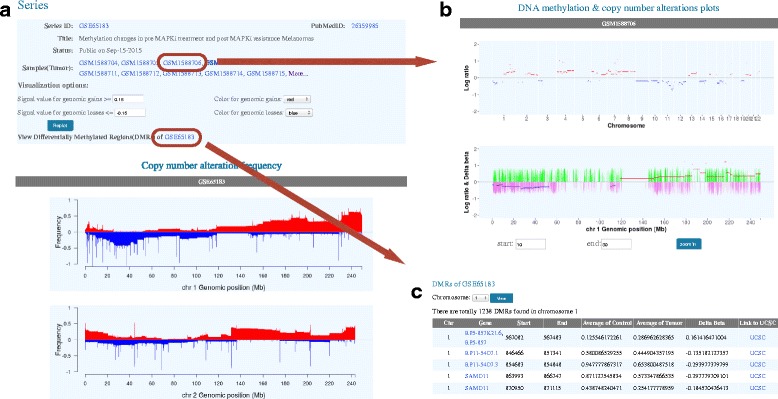
An example of GEO data series browsing. **a** The copy number gain and loss frequencies for each chromosome across the data series (only chromosome 1 and 2 are shown here). **b** Illustration of sample-level data. **c** The annotations of the differentially methylated regions and the associated genes

MethCNA also provides data browsing interface to allow researchers to investigate mutation profiles of each study. The ‘Browse’ page contains all MethCNA data organized by studies. Clicking on the article title leads to a page that includes a table with detailed information of the dataset and genome-wide frequency plots of copy number alterations across all samples in the study (Fig. 4a). The frequency plots for each chromosome provide an intuitive view of regional chromosomal rearrangement hotspots. The user can also access sample-level data by clicking on the link for each array ID (Fig. 4b). The result page contains a link to view the annotations of the differentially methylated regions (DMR), as well as detailed information about the genes that overlap with these regions (Fig. 4c). The DMR page also generates links to UCSC Genome Browser [30] and COSMIC database [33] for further annotation.

### Data clustering

**Fig. 5:**
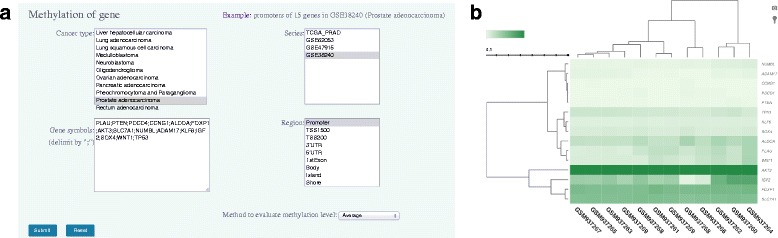
An example of hierarchical clustering of DNA methylation data. **a** The interface for user to input gene symbols and select cancer type, data series and gene regions. **b** The clustering diagram shows the methylation profile of queried genes among the selected samples. The specific region can be zoomed in for an enlarged view

Most tumors display genome-wide abnormal genetic and epigenetic events, which provide their genomes with complex mutational patterns. Hierarchical clustering is a widely used method for large-scale genomic data analysis. The ‘Cluster’ page of MethCNA provides clustering of copy number alterations and DNA methylation data for users to characterize different aberration patterns in data series. The clustering of CNA and methylation data will be investigated separately. For CNA data, the users are able to investigate tumor type specific datasets. The data series can be chosen in the list box according to cancer types. In the clustering graph, tumor samples with similar CNA profiles are grouped together. The regional hotspots can be seen intuitively, and it is particularly helpful when comparing CNA profiles across multiple datasets. Investigation of these hotspots has proven to be an effective methodology to identify novel cancer genes. Furthermore, the identified clusters may represent distinct cancer subtypes. For DNA methylation profiles, we developed gene-level clustering analysis. The user can input a list of gene symbols and specify cancer type and gene region to run the analysis (Fig. 5a). The result page will return a clustering diagram to show the methylation profile of queried genes among the selected samples (Fig. 5b). The interactive diagram can be zoomed in for more detailed information. It enables researchers to investigate the synergistic effect of aberrant methylation of multiple genes in tumorigenesis.

### Case study

**Fig. 6:**
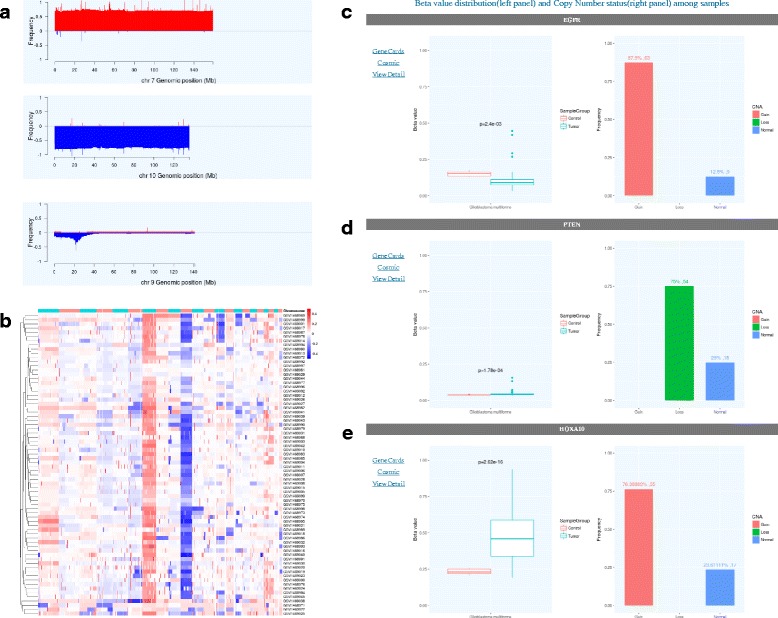
An example of glioblastoma data analysis. **a** The copy number frequency plots exhibit the whole chromosome 7 gain, whole chromosome 10 loss and a focal deletion on chromosome 9p. **b** Heatmap of copy number profiles across 72 glioblastoma samples. **c** EGFR promoter hypomethylation and copy number gain are shown by boxplot and histogram, respectively. **d** PTEN promoter hypermethylation and copy number loss. **e** HOXA10 promoter hypermethylation and copy number gain

As an illustration of MethCNA functionalities, we analyzed two glioblastoma (GBM) datasets that were integrated into our database: GSE60274 and TCGA-GBM. There are 72 and 153 tumor samples in GEO and TCGA datasets, respectively. In the ‘Browse’ page, clicking on the series title of GSE60274 opens a result page with basic information of the dataset. It also contains histograms of each chromosome representing the CNA frequencies. The genome-wide profile of chromosomal alteration hotspots may point to genomic loci harbouring cancer related genes. We found that the whole chromosome 7 gain and chromosome 10 loss were the most prevalent genomic imbalances in this dataset (Fig. 6a). According to recent studies, these alterations are the evolutionary first driver events in the development of primary GBM [34, 35]. Furthermore, we observed a recurrent focal deletion of the short arm of chromosome 9 (Fig. 6a). The peak region of focal deletion on chromosome 9p encompassing the tumor suppressor gene CDKN2A, which may play a crucial role to promote GBM progression [36, 37]. CDKN2A has been reported as an indicator of poor prognosis and is hypothesized to be a cancer driver gene [38]. The similar CNA frequency profile was observed in the TCGA-GBM dataset (Additional file 3: Figure S1).

The heatmaps of both datasets can be inspected on the ‘Cluster’ page. For example, we selected ‘Glioblastoma’ and ‘GSE60274’ in the ‘Cancer type’ and ‘Series’ list box, respectively. The overall CNA profile of the 72 tumor samples in GSE60274 was shown. The high prevalence of whole chromosome 7 and 10 abnormalities can be clearly seen (Fig. 6b). The distribution of focal aberrations can also be explored. For the TCGA-GBM dataset, we got the similar overall pattern of results (Additional file 4: Figure S2). The specific pattern of genomic copy number profiles indicates the non-random occurrence of CNAs, and may provide important information for understanding the molecular mechanisms involved in tumorigenesis.

We also investigated the synergistic effects of genomic aberrations and epigenetic changes in GSE60274. In the ‘query by genes’ interface of ‘Search’ page, we searched for three genes that are implicated in cancer initiation or progression: EGFR, PTEN and HOXA10. We selected ‘Glioblastoma’ and ‘GSE60274’ in ‘Cancer type’ and ‘Series’ list box, respectively. In the ‘Gene symbols’ text field, we input the above gene names separated by semicolon. For the gene region, we selected ‘Promoter’ in the drop-down list for analysis. In the results page, box plot and histogram were generated to present DNA methylation and copy number status for each gene across glioblastoma samples. According to the results, EGFR promoter region was significantly hypomethylated (*P* < 0.01, two-sided t test) in glioblastoma, as compared with normal brain specimens (Fig. 6c). Simultaneously, the copy number of EGFR was increased in most samples (63 out of 72 samples, 87.5%). EGFR is a transmembrane tyrosine kinase receptor that plays a central role in regulating cell proliferation and differentiation. Overexpression of EGFR has been reported and implicated in the pathogenesis of many cancer types [39, 40]. On the contrary, the tumor suppressor gene PTEN was inactivated by hypermethylation (*P* < 0.001, two-sided t test) and genomic loss (54 out of 72 samples, 75%) throughout the dataset (Fig. 6d). The genomic and epigenomic alterations affecting the expression of PTEN may indicate Knudson’s ‘two-hit’ hypothesis in tumorigenesis [41], and were reported to be crucial driver events in glioblastoma initiation and progression [42, 43]. Interestingly, the HOXA10 gene was found to be hypermethylated (*P* < 10–16, two-sided t test) and showed copy number gain (55 out of 72 samples, 76%) in the dataset (Fig. 6e). It revealed the theory that gene dosage may contribute to the aberrant gene expression, as reported recently by Kurscheid et al. [44]. Our results demonstrate the utility of MethCNA in integrating genomic and epigenomic alteration data and the ability to identify cancer driver genes.

## Conclusion

MethCNA is an effort to further our understanding of the relationship between copy number alterations and DNA methylation status, both of which are known to be hallmarks of human cancer. There are several publicly available resources similar to our database. The cBioPortal [45] is a resource for interactive exploration of multidimensional cancer genomics data sets, including CNA and DNA methylation data. CNAmet [46] is an R package for integrative analysis of high-throughput copy number, DNA methylation and gene expression data. However, these tools were designed to integrate data derived from different patients or platforms. The most important advantage of our database is that the methylation and copy number data are derived from exactly the same genomic loci. As previous studies have indicated, genetic and epigenetic alterations may act coordinately to fulfil the two-hit paradigm at a gene-specific level, leading to cancer initiation. For example, tumor suppressor genes that undergo a ‘double hit’, such as heterozygous loss and hypermethylation, or oncogenes in an amplified region that are hypomethylated are most likely to be drivers of tumorigenic processes. Thus, the efficient mining of this large-scale dataset can provide valuable insight into the underlying molecular bases of oncogenesis, and facilitate to distinguish driver from passenger alterations. We believe that our database is a powerful tool not only for bioinformaticians but also for experimental researchers. Concerning the future development of our database, we plan to control variations in the tumor cell content between samples. There are several tools developed for the correction of cell type heterogeneity and control for false positives in large-scale epigenome data analysis, such as ReFACTor [47] and MeDeCom [48]. In the next release of our database, we will utilize these tools to remove confounding variation and to provide a better framework for data interpretation.

## Additional files

Additional file 1: **Table S1.** List of GEO tumor samples that are integrated in MethCNA. (XLS 290 kb)

Additional file 2: **Table S2.** List of TCGA samples that are integrated in MethCNA. (XLS 1181 kb)

Additional file 3: **Figure S1.** The genome-wide copy number aberration frequency profile of the TCGA GBM dataset. (PDF 1661 kb)

Additional file 4: **Figure S2.** The copy number heatmap for 153 glioblastoma samples of the TCGA GBM dataset. (PDF 438 kb)

## Abbreviations

450 k: Illumina Infinium HumanMethylation450 BeadChip
CGI: CpG island
CNAs: Copy number aberrations
COSMIC: Catalogue of Somatic Mutations in Cancer
DMP: Differentially methylated positions
DMR: Differentially methylated regions
GBM: Glioblastoma
GEO: Gene Expression Omnibus
MDPE: Maximum density peak estimation
SNP: Single nucleotide polymorphism
TCGA: The Cancer Genome Atlas
TSS: Transcriptional start site
UTR: Untranslated region
