# Supplementary figures and images for: MethCNA: a database for integrating genomic and epigenomic data in human cancer
Source: BMC Genomics. 2018 Feb 13;19:138. doi: 10.1186/s12864-018-4525-0 (PMC5810021; doi:10.1186/s12864-018-4525-0)

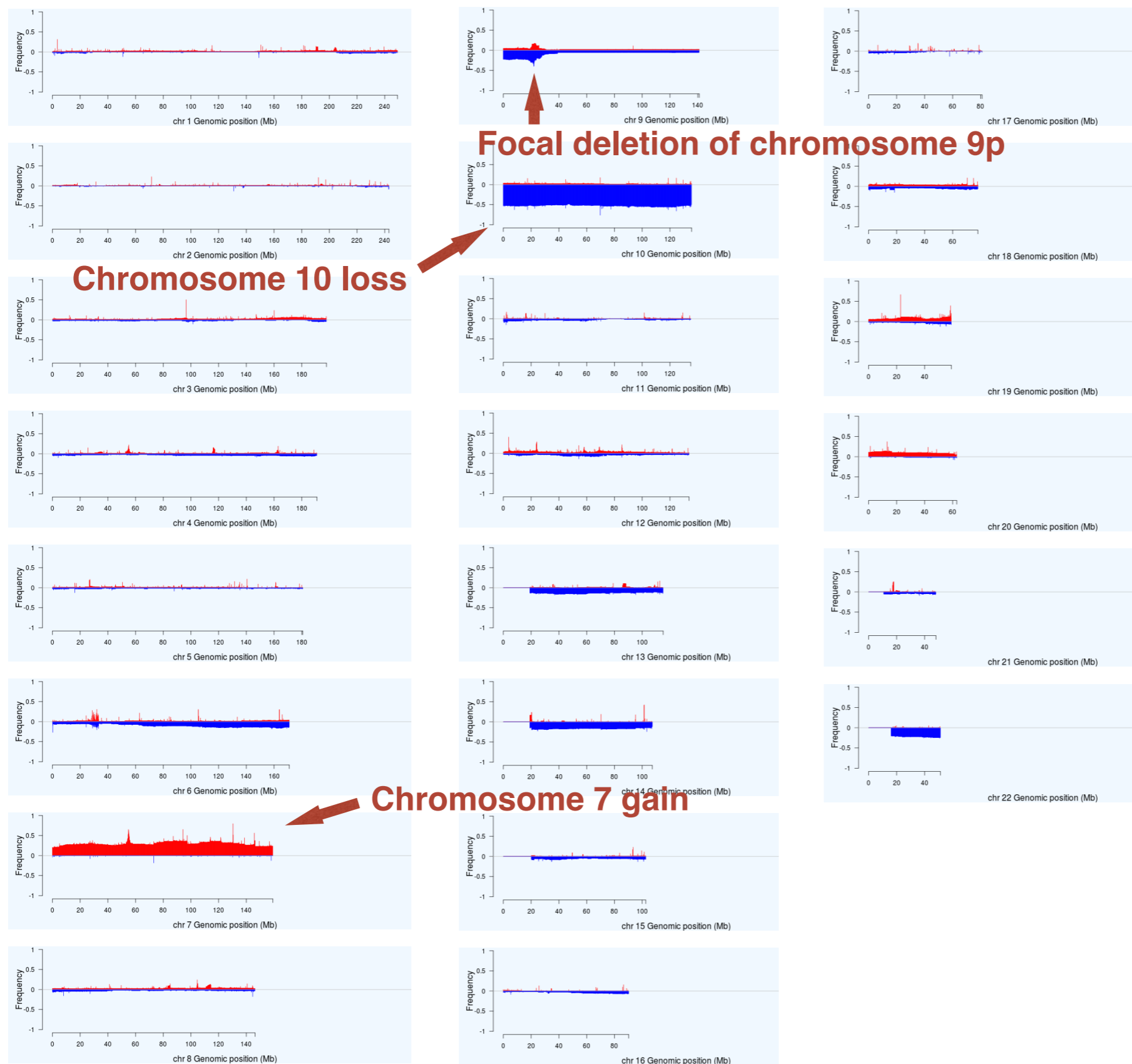

**Figure S1.** The genome-wide copy number aberration frequency profile of the TCGA GBM dataset.

Supplement: Supplementary file 3 — Figure S1. The genome-wide copy number aberration frequency profile of the TCGA GBM dataset. (PDF 1661 kb) [file 12864_2018_4525_MOESM3_ESM.pdf]

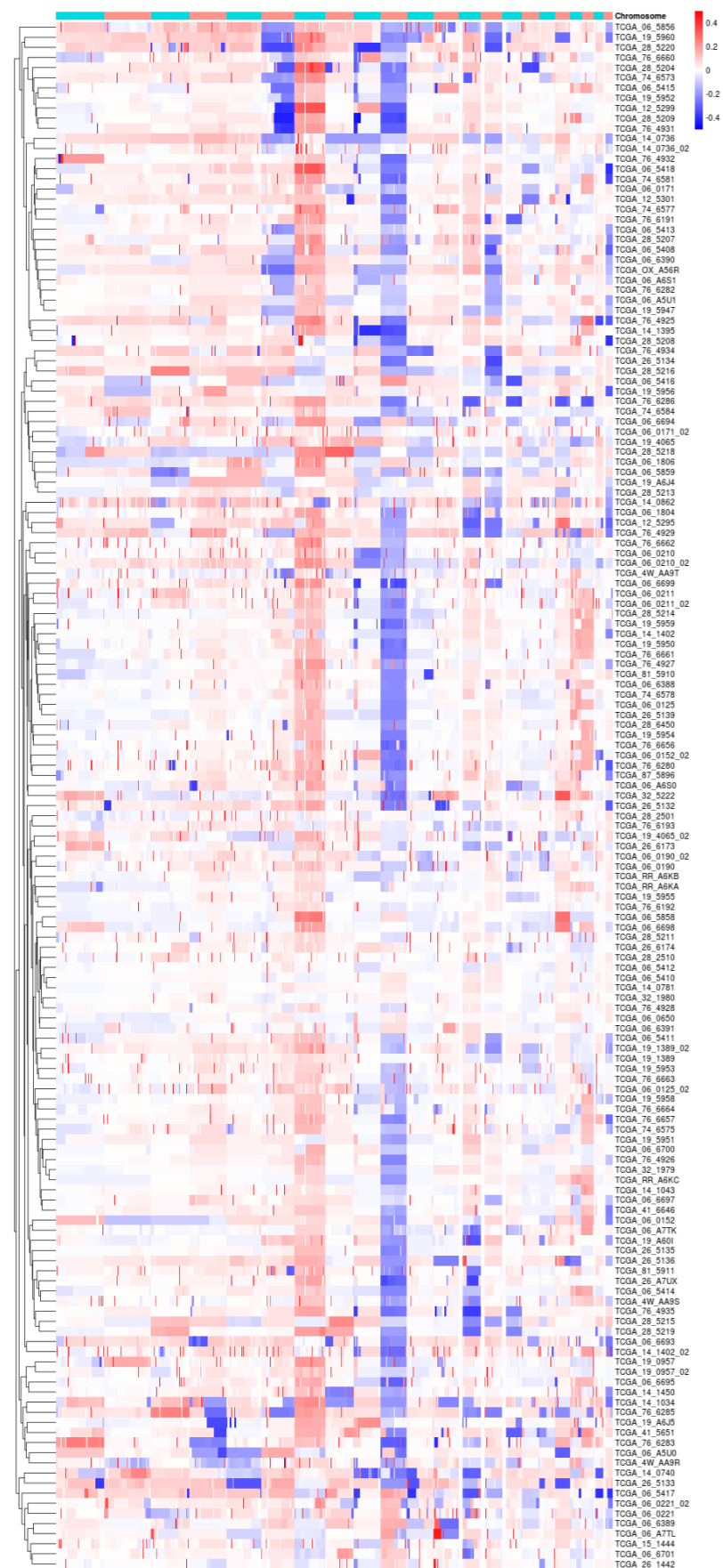

**Figure S2.** The copy number heatmap for 153 glioblastoma samples of the TCGA GBM dataset.

Supplement: Supplementary file 4 — Figure S2. The copy number heatmap for 153 glioblastoma samples of the TCGA GBM dataset. (PDF 438 kb) [file 12864_2018_4525_MOESM4_ESM.pdf]
